# Diagnostic performance of 1-hour plasma glucose and glucose curve shape during oral glucose tolerance test: a cross-sectional study in a Brazilian cohort

**DOI:** 10.1186/s13098-025-01977-1

**Published:** 2025-11-14

**Authors:** João Pedro F. C. Castro, Delanie B. Macedo, Rejane A. Magalhaes, Cecília M. M. Figueirêdo, Rachel Petrola, Milena G. Teles

**Affiliations:** 1 Faculty of Medicine of the University of Fortaleza and Medical Intern at Emilio Ribas Medicina Diagnostica Ltda, Fortaleza, Ceará Brazil; 2 Emilio Ribas Medicina Diagnostica Ltda, Av. Barão de Studart, 730, Fortaleza, Ceará CEP: 60120000 Brazil

**Keywords:** Oral glucose tolerance test, 1-h glucose, 2-h glucose, Glucose curve morphology, Diagnostic performance, Impaired glucose tolerance, Diabetes diagnosis

## Abstract

**Background:**

The 1-h plasma glucose (1-h PG) during oral glucose tolerance test (OGTT) may enhance early dysglycemia detection compared with conventional markers. Distinct glucose curve morphologies correlate with glycemic profiles, insulin sensitivity, and β-cell function. We aimed to compare 1-h PG diagnostic performance with conventional methods and explored glucose curve morphology prevalence in a Brazilian population.

**Methods:**

This retrospective cross-sectional study analyzed 1,797 OGTT records from a Brazilian laboratory (2021–2024). Glycemic profiles were classified using 1-h PG, fasting plasma glucose (FPG), 2-h PG, and HbA1c according to American Diabetes Association, Brazilian Diabetes Society and International Diabetes Federation criteria. Cohen's κ assessed inter-method agreement, McNemar’s chi-square tests compared high glycemic risk detection and χ^2^ tests evaluated associations between curve patterns (monophasic, biphasic, continuous rise) and glycemic status.

**Results:**

Participants were predominantly female (68.9%; mean age 49.6 ± 15.0 years). The 1-h PG identified 27.8% more intermediate hyperglycemia (IH)/type 2 diabetes mellitus (T2DM) cases than FPG, 16.8% more than 2-h PG, and 26.7% more than HbA1c. The 1-h PG identified more individuals at high glycemic risk than FPG (χ^2^ = 290, p < 0.001), HbA1c (χ^2^ = 129, p < 0.001), 2-h PG (χ^2^ = 165, p < 0.001), and their combined criteria. Among 1361 evaluable curves, 59.6% were monophasic, 38.3% biphasic, and 2.1% continuous rise**.** Monophasic curves predominated in IH/T2DM, whereas biphasic curves were more frequent in normoglycemia (χ^2^ = 278; P < 0.001).

**Conclusions:**

The 1-h PG thresholds ≥ 8.6 mmol/L (155 mg/dL) for IH and ≥ 11.6 mmol/L (209 mg/dL) for T2DM identified more dysglycemia cases than conventional methods, supporting early detection utility. Monophasic curve morphology was associated with impaired glycemic profiles, highlighting prognostic relevance.

## Background

Diabetes mellitus (DM) is a highly prevalent chronic disease worldwide. Type 2 diabetes mellitus (T2DM) is characterized by chronic hyperglycemia resulting from progressive insulin resistance and pancreatic β-cell dysfunction. T2DM accounts for approximately 90% of all diabetes cases, affecting an estimated 589 million individuals globally and 16.6 million in Brazil as of 2024 [1].

Intermediate hyperglycemia (IH), or prediabetes, represents a metabolic state in which blood glucose concentrations exceed normal ranges but remain below T2DM diagnostic thresholds [2, 3]. According to the American Diabetes Association (ADA) and Brazilian Diabetes Society (SBD), IH is defined by fasting plasma glucose (FPG) concentrations of 5.6–6.9 mmol/L (100–125 mg/dL) (impaired fasting glucose -IFG), 2-h plasma glucose (2-h PG) during an oral glucose tolerance test (OGTT) of 7.8–11.0 mmol/L (140–199 mg/dL) (impaired glucose tolerance-IGT), or glycated hemoglobin (HbA1c) of 5.7%–6.4%. The World Health Organization (WHO) defines IGT as 2-h PG of 7.8–11.0 mmol/L (140–199 mg/dL) and IFG as FPG of 6.1–6.9 mmol/L (110–125 mg/dL) [1, 4]. Individuals with IH face elevated risks for developing T2DM and cardiovascular disease; however, early intervention—whether through lifestyle-based or pharmacologic—can reverse this condition [2, 3, 5].

Early detection of IH and T2DM is crucial for timely intervention and prevention of dysglycemia progression and its associated complications, including retinopathy, diabetic kidney disease, and peripheral neuropathy [2, 3]. However, some clinical conditions render conventional diagnostic methods less effective for early detection. Notably, 4 in 10 individuals with diabetes remain undiagnosed [1, 6, 7]. Furthermore, the absence of standardized criteria for classifying IH also delays diagnosis and contributes to the increasing prevalence of prediabetes and diabetes [6, 8].

In this context, the International Diabetes Federation (IDF) published guidelines in 2024 supporting the use of 1-h plasma glucose (1-h PG) values during the OGTT—defined as ≥ 8.6 mmol/L (155 mg/dL) for IH identification and ≥ 11.6 mmol/L (209 mg/dL) for T2DM diagnosis—as a highly sensitive method for early dysglycemia detection. Studies have shown that in diverse populations, 1-h PG ≥ 8.6 mmol/L (155 mg/dL), regardless of IGT, IFG, or HbA1c ≥ 5.7%, status, is strongly predictive of T2DM progression [9–12], and may detect metabolic abnormalities earlier than other conventional markers [10, 13, 14], which are often associated with adverse outcomes. Elevated 1-h PG concentrations are also associated with hyperglycemia-related complications such as progressive pancreatic β-cell dysfunction, cardiovascular and renal disease, retinopathy, and increased mortality [10, 15–17].

Moreover, analysis of additional OGTT curve-derived data provides clinically meaningful information that may guide clinical decision-making and improve patient prognosis. The shape of the OGTT glucose curve reflects pancreatic β-cell function. A monophasic curve—characterized by a single glucose peak followed by a decline of ≥ 0.25 mmol/L (4.5 mg/dL) —compared to a biphasic curve, which includes two glucose peaks, is associated with: a. reduced peripheral insulin sensitivity; b. impaired β-cell insulin secretion; and c. increased risk of T2DM development. Furthermore, the most adverse prognostic pattern is the rising curve, defined by the absence of any identifiable peak [18–20].

The main objective of this study is to compare the diagnostic performance 1-h PG in classifying glycemic profiles as normal glucose tolerance (NGT), IH, or diabetes, with the performance of HbA1c, FPG, and 2-h PG in the Brazilian population. Additionally, as a secondary aim, the study seeks to investigate the prevalence of OGTT curve patterns within the studied sample.

## Methods

### Study design and participants

This retrospective, observational, cross-sectional study included patients who underwent the OGTT at the Emilio Ribas Medicina Diagnostica Ltda, located in northeastern Brazil, between 2021 and 2024. Data collection was conducted from December 2024 to June 2025, yielding an initial dataset of 1,921 records of non-pregnant patients. Patients under 18 years of age and those receiving oral or injectable antidiabetic medications were excluded from the analysis.

A total of 1797 eligible patient records were analyzed to compare the diagnostic performance of 1-h PG with FPG and 2-h PG. For comparisons involving HbA1c, an additional 953 patients were excluded due to the absence of HbA1c measurements.

For the analysis of OGTT glucose curve morphology, data from 436 patients who had only 3- (0, 60 and 120 min) or 4- (0, 60, 90 and 120 min) point sampling during the OGTT were excluded, retaining only those with 5- (0, 30, 60, 90 and 120 min), 6- (0, 30, 60, 90 and 120 and 150 min), or 7- (0, 30, 60, 90 and 120, 150 and 180 min) point glucose measurements for curve shape analysis.

All data were obtained from the laboratory's digital information system and handled in accordance with Brazilian ethical standards, under approval number: 85521024.5.0000.0229.

### Clinical and laboratory data

Clinical data collected from the participants included age and sex. Laboratory measurements encompassed FPG, HbA1c, 1-h PG, and 2-h PG values obtained through the OGTT. The OGTT was performed after a minimum of 8 h of fasting, using a standardized 75 g glucose load. Blood samples were collected at multiple time points—ranging from 3 to 7—depending on the physician's request for each individual case. Plasma glucose measurements were performed using the hexokinase method (Siemens), and HbA1c levels were determined by high-performance liquid chromatography (HPLC-Bio-Rad) using an National Glycohemoglobin Standardization Program-certified methodology.

The primary outcome of this study was the classification of the participants' glycemic profiles into three clinical categories: NGT, IH, and DM. This classification followed the diagnostic criteria established by the ADA and SBD, in which NGT is defined by FPG levels below 5.6 mmol/L (100 mg/dL), 2-h PG concentrations below 7.8 mmol/L (140 mg/dL), and HbA1c below 5.7%; IH is defined by FPG between 5.6–6.9 mmol/L (100–125 mg/dL) (IFG), 2-h PG between 7.8–11.0 mmol/L (140–199 mg/dL) (IGT), and HbA1c between 5.7% and 6.4%; and DM is defined by FPG equal to or greater than 7.0 mmol/L (126 mg/dL), 2-h PG equal to or greater than 11.1 mmol/L (200 mg/dL), and HbA1c equal to or greater than 6.5% [4, 5]. For the classification based on 1-h PG, the criteria proposed by the IDF and SBD were applied, which define NGT as 1-h PG below 8.6 mmol/L (155 mg/dL), IH as 1-h PG 8.6–11.5 mmol/L (155—208 mg/dL), and DM as 1-h PG equal to or greater than 11.6 mmol/L (209 mg/dL) [5, 10]. Furthermore, for each diagnostic method, individuals were considered to have high glycemic risk when they met the criteria for either IH or DM.

### Curve shape classification

Regarding the glucose curve shape classification, a monophasic pattern was defined as a continuous rise in plasma glucose concentration reaching a single peak, followed by a subsequent decrease of more than 0.25 mmol/L (4.5 mg/dL). A biphasic pattern was characterized by an initial rise and fall in glucose concentration concentrations, followed by a secondary increase of greater than 0.25 mmol/L (4.5 mg/dL) after the initial decline. A continuous or rising pattern was defined as a curve without identifiable peaks, exhibiting a persistent upward trajectory in glucose levels throughout the test duration [20].

### Statistical analysis

Statistical analyses were performed using Jamovi software, version 2.23.28.0. Continuous variables were expressed as means and standard deviations (SD), whereas categorical variables were presented as absolute frequencies and proportions. Cohen’s Kappa k coefficient was employed to assess the agreement between 1-h PG and the other diagnostic tests regarding glycemic classification (primary outcome). The diagnostic capacity to identify individuals at high glycemic risk (IH or T2DM) was compared across tests. Specifically, 1-h PG was contrasted with FPG, HbA1c, and 2-h PG, using McNemar’s chi-square test for paired categorical data. This approach evaluates whether the frequency of discordant classifications between tests is significantly different within the same individuals. In addition, we also compared the detection capacity of 1-h PG with the combined use of FPG plus HbA1c, 2-h PG plus FPG, and the use of FPG, HbA1c, and 2-h PG together. The chi-square (χ^2^) test was used to compare the distribution of OGTT glucose curve patterns across different metabolic profiles as determined by 1-h PG classification. In addition to the primary analyses, we performed stratified comparisons to explore potential differences by age and sex. For age-stratified analyses, we used 35 years as the cutoff, in accordance with the SBD, which considers individuals aged ≥ 35 years at increased risk for developing diabetes. [5] Diagnostic concordance between 1-h PG and standard tests (FPG, 2-h PG, HbA1c) was assessed separately for participants < 35 years and ≥ 35 years. Similarly, sex-stratified analyses were performed to evaluate differences in agreement patterns between tests and in the association between glycemic status and OGTT curve shape. Chi-square tests (χ^2^) were applied for categorical comparisons, with statistical significance set at p < 0.05.

## Results

### Baseline characteristics according to glycemic profile

A total of 1797 OGTT records were included in the analysis. Most participants were female (68.9%), with a mean age of 49.6 ± 15.0 years. The mean FPG was 5.02 ± 0.62 mmol/L (90.3 ± 11.2 mg/dL), mean HbA1c was 5.23 ± 0.39%, mean 1-h PG was 7.99 ± 2.46 mmol/L (144.0 ± 44.4 mg/dL), and mean 2-h PG was 6.72 ± 2.38 mmol/L (121.0 ± 42.9 mg/dL) (Table 1).

**Table 1:** Participant characteristics at baseline examination according to glycemic profile

	n	
**Sex**	1797	
Female		1239 (68.9%)
Male		558 (31.1%)
**Age**	1797	
18–30		149 (8.3%)
30–60		1133 (63.0%)
60–80		485 (27%)
> 80		30 (1.7%)
**FPG**	1797	5.0 ± 0.6 mmol/L (90.3 ± 11.2 mg/dL)
NGT		1514 (84.3%)
IFG		266 (14.8%)
Diabetes		17 (0.9%)
**1-h PG**	1797	8.0 ± 2.5 mmol/L (144 ± 44.4 mg/dL)
NGT		1121(62.4%)
IH		538 (29.9%)
Diabetes		138 (7.7%)
**2-h PG**	1797	6.7 ± 2.4 mmol/L (121 ± 42.9 mg/dL)
NGT		1365 (76%)
IGT		327 (18.2%)
Diabetes		105 (5.8%)
**HbA1c**	844	5.23 ± 0.394%
NGT		735 (87.1%)
Prediabetes		104 (12.3%)
Diabetes		5 (0.6%)

*FPG* fasting plasma glucose, *NGT* normal glucose tolerance, *IFG* impaired fasting glucose; 1-H PG, 1 h plasma glucose; IH, intermediate hyperglycemia; *IGT* impaired glucose tolerance; *2-H PG* 2 h plasma glucose, *HbA1c* glycated hemoglobin

### Comparison between the diagnostic performance of the tests

**Fig. 1:**
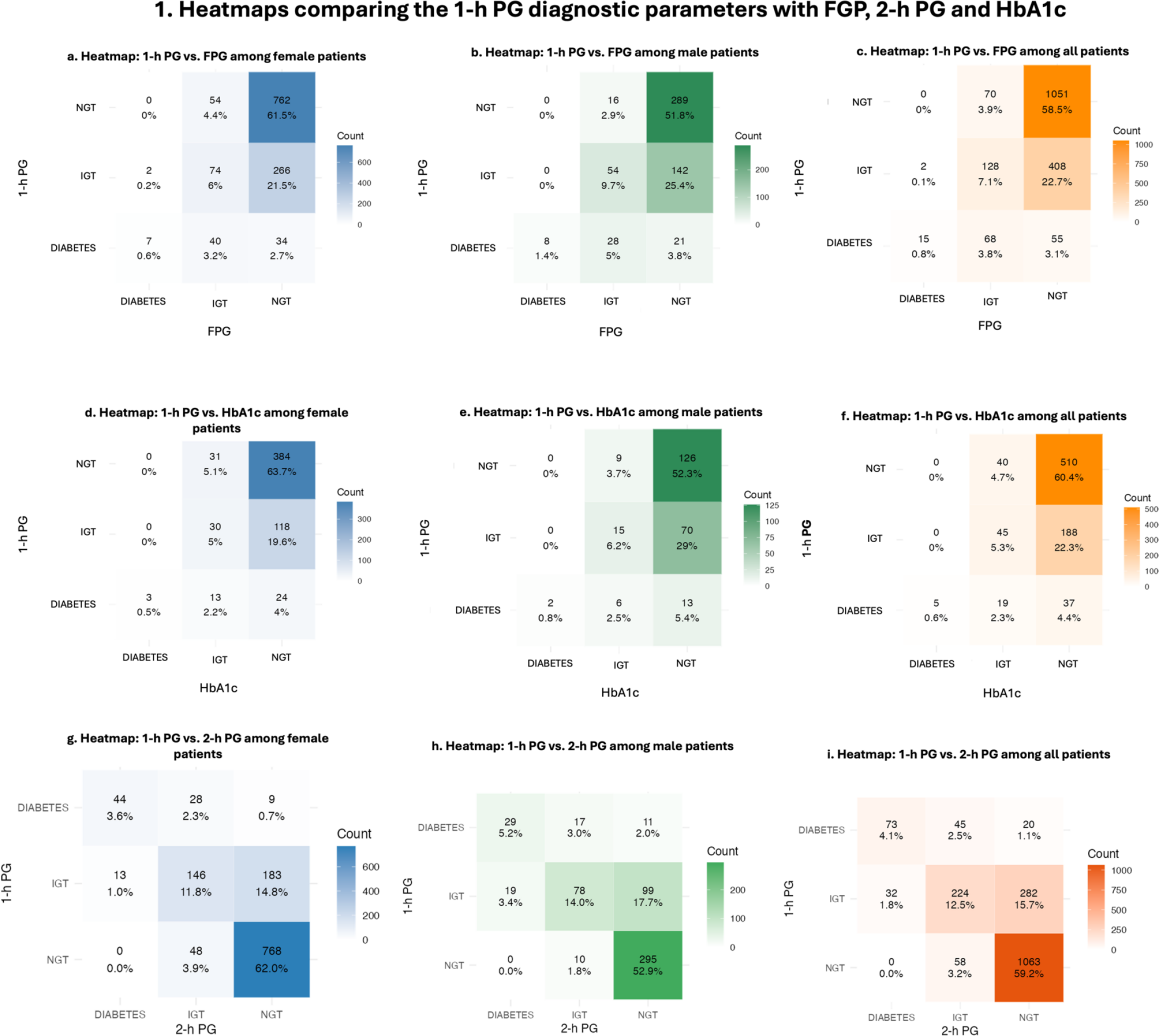
Heatmaps comparing the 1-h PG diagnostic parameters with FPG, 2-h PG, and HbA1c. Figure 1A. The 1-h PG vs. FPG comparison in women showed significant discrepancies (χ²=224; df=4; N=1239). Figure 1B. The 1-h PG vs. FPG comparison in men showed significant discrepancies (χ²=163; df=4; N=558). Figure 1C. The 1-h PG showed fair agreement with FPG in all patients (κ=0.219, P<0.001). Figure 1D. The 1-h PG vs. HbA1c comparison in women showed greater discrepancies (χ²=76.8; df=4; N=603). Figure 1E. The 1-h PG vs. HbA1c comparison in men showed lower discrepancies (χ²=33.2; df=4; N=241). Figure 1F. The 1-h PG showed poor agreement with HbA1c in all patients (κ=0.155, P<0.001). Figure 1G. The 1-h PG vs. 2-h PG comparison in women showed the strongest discrepancies (χ²=769; df=4; N=1239). Figure 1H. The 1-h PG vs. 2-h PG comparison in men also showed significant discrepancies (χ²=294; df=4; N=558). Figure 1I. The 1-h PG showed moderate agreement with 2-h PG in all patients (κ=0.479, P<0.001). 1-h PG, 1-hour plasma glucose; FPG, fasting plasma glucose; 2-h PG, 2-hour plasma glucose; HbA1c, glycated hemoglobin; NGT, normal glucose tolerance; IH, intermediate hyperglycemia; T2DM, type 2 diabetes mellitus.

Diagnostic concordance between 1-h PG and the other glycemic markers was assessed using Cohen’s Kappa k coefficient. The agreement between 1-h PG and FPG was 66.4%, with a Kappa k value of 0.219, indicating fair agreement (P < 0.001). In this comparison, the 1-h PG identified 463 additional cases (27.8%) of IH or T2DM not detected by FPG. The agreement between 1-h PG and 2-h PG was 75.7%, with a Kappa k coefficient of 0.479, representing moderate agreement (P < 0.001); the 1-h PG detected 302 additional cases (16.8%) of IH or T2DM. When compared with HbA1c, 1-h PG demonstrated a concordance rate of 66.3%, with a Kappa k of 0.155, reflecting poor agreement (P < 0.001), and identified 225 additional cases (26.7%) of IH or T2DM. (Fig. 1).

**Fig. 2:**
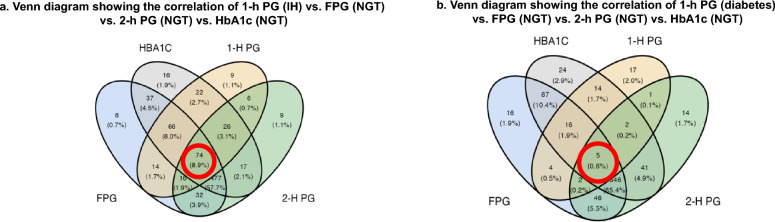
Venn Diagrams comparing the 1-h PG diagnostic parameters with FPG, 2-h PG and HbA1c simultaneously. Legend: **A**. The 1-h PG identified 8.9% additional individuals with IH not detected by FPG, 2-h PG, or HbA1c. **B**. The 1-h PG identified 0.6% additional individuals with T2DM not detected by the other tests. 1-h PG, 1-h plasma glucose; 2-h PG, 2-h plasma glucose; IH, intermediate hyperglycemia; NGT, normal glucose tolerance; T2DM, type 2 diabetes mellitus

When the four diagnostic methods (FPG, 2-h PG, HbA1c, and 1-h PG) were evaluated simultaneously, concordance across all tests occurred in 57.0% of the cases, with a Kappa k coefficient of 0.251, indicating fair agreement (P < 0.001). Notably, the 1-h PG test identified IH in 8.9% and diabetes in 0.6% of individuals who were otherwise classified as having NGT by FPG, 2-h PG, and HbA1c. (Fig. 2).

When comparing the diagnostic classification of 1-h plasma glucose with standard markers, significant discrepancies were observed across age groups. For the 2-h PG, discordance was detected in both age groups—individuals aged ≥ 35 years (χ^2^ = 874.7, df = 4, p < 0.001) and < 35 years (χ^2^ = 85.2, df = 4, p < 0.001)- although the disagreement was markedly greater in the older group. For FPG, a significant difference was found in participants ≥ 35 years (χ^2^ = 339, df = 4, p < 0.001) and in the overall sample (χ^2^ = 401, df = 4, p < 0.001). However, comparison in the < 35 year-group was not feasible due to the very low number of cases identified by FPG criteria. A similar pattern was seen with HbA1c: significant discordance was found in the ≥ 35- year group (χ^2^ = 91.8, df = 4, p < 0.001) and in the overall sample (χ^2^ = 110.3, df = 4, p < 0.001), but not among those < 35 years owing to the insufficient case numbers. These findings suggest that 1-h PG classifies glycemic risk differently compared to 2-h PG, FPG, and HbA1c, especially among individuals aged ≥ 35 years. In younger participants, the low number of cases limited meaningful comparison for FPG and HbA1c.

When stratified by sex, the comparison between 1-h PG and conventional diagnostic methods (FPG, HbA1c, and 2-h PG) revealed significant discrepancies in both men and women (all p < 0.001). The magnitude of these divergences, however, was consistently greater among women. For FPG, the association between classifications yielded χ^2^ = 224 with df = 4 (N = 1239) in women and χ^2^ = 163 with df = 4 (N = 558) in men. For HbA1c, the discrepancies were also higher in women (χ^2^ = 76.8; df = 4; N = 603) compared with men (χ^2^ = 33.2; df = 4; N = 241). The strongest divergences were observed in relation to 2-h PG, with χ^2^ = 769 (df = 4; N = 1239) in women versus χ^2^ = 294 (df = 4; N = 558) in men. Taken together, these results demonstrate that although the 1-h PG test significantly diverges from traditional diagnostic methods in both sexes, the degree of disagreement is systematically more pronounced in women (Fig. 1).

### Comparison in the detection of individuals at high glycemic risk

When comparing the diagnostic classification of high glycemic risk across tests, significant differences were observed between 1-h PG and all other glycemic parameters. Against FPG, the McNemar test indicated a higher proportion of individuals identified as high risk by 1-h PG (1-h PG: 37,6%; FPG: 15,7%; χ^2^ = 290, p < 0.001). Similarly, when compared with HbA1c, 1-h PG classified substantially more individuals as high risk (1-h PG: 34,8%; HbA1c: 12,9%; χ^2^ = 129, p < 0.001). In the combined analysis of FPG or HbA1c, the discordance remained significant in favor of 1-h PG (1-h PG: 34,8%; FPG or HbA1c = 22,5%; χ^2^ = 49.6, p < 0.001). Finally, when compared with 2-h PG, 1-h PG again identified a significantly greater number of individuals at high risk (1-h PG: 37,6%; 2-h PG: 24%; χ^2^ = 165, p < 0.001). In analyses using broader combined criteria, 1-h PG remained significantly different, detecting more individuals than the composite of FPG or 2-h PG (1-h PG: 37,6%; FPG or 2-h PG: 31,2%; χ^2^ = 37.0, p < 0.001) and then the combination of FPG, HbA1c, or 2-h PG (1-h PG: 37,6%; FPG, HbA1c or 2-h PG: 33,9%; χ^2^ = 151, p < 0.001). Overall, these findings consistently demonstrate that 1-h PG classified more individuals as having high glycemic risk than the other conventional glycemic parameters.

### Curve shape analysis

Among the 1,361 OGTT eligible for curve analysis, 59.6% of the curves were classified as monophasic (M), 38.3% as biphasic (B), and 2.1% as continuous increase (C). Biphasic curves exhibited the lowest mean values and variability (mean ± SD): FPG, 4.88 ± 0.50 mmol/L (87.9 ± 8.99 mg/dL); HbA1c, 5.17 ± 0.355%; 1-h PG, 6.38 ± 1.88 mmol/L (115 ± 33.8 mg/dL); and 2-h PG, 5.77 ± 1.67 mmol/L (104 ± 30 mg/dL). Curves with continuous increase presented the highest mean values and variability: FPG, 5.36 ± 0.72 mmol/L (96.5 ± 12.9 mg/dL); HbA1c, 5.40 ± 0.637%; 1-h PG, 10.27 ± 2.05 mmol/L (185 ± 37.0 mg/dL); and 2-h PG, 11.66 ± 2.49 mmol/L (210 ± 44.8 mg/dL). Monophasic curves demonstrated intermediate values: FPG, 5.10 ± 0.61 mmol/L (91.8 ± 10.9 mg/dL); HbA1c, 5.24 ± 0.368%; 1-h PG, 8.88 ± 2.31 mmol/L (160 ± 41.7 mg/dL); and 2-h PG, 7.10 ± 2.44 mmol/L (128 ± 44 mg/dL). (Table 2).

**Table 2:** Participant characteristics at baseline examination according to OGTT curve shape

	Monophasic^*a*^	Biphasic^*b*^	Continuous^*c*^
*n*	811 (59.6%)	521 (38.3%)	29 (2.1%)
Sex			
Female	519 (38.1%)	379 (27.8%)	19 (1.4%)
Male	292 (21.5%)	142 (10.4%)	10 (0.7%)
Age (y)	51.2 ± 14.7	48.6 ± 14.3	62 ± 17.1
18–30	53 (3.9%)	46 (3.4%)	2 (0.1%)
30–60	501 (36.8%)	349 (25.6%)	8 (0.6%)
60–80	239 (17.6%)	122 (9%)	16 (1.2%)
> 80	18 (1.3%)	4 (0.3%)	3 (0.2%)
1-h PG			
NGT	375 (27,6%)	467 (34.3%)	7 (0.5%)
IGT	346 (25.4%)	48 (3.5%)	15 (1.1%)
Diabetes	90 (6.6%)	6 (0.4%)	7 (0.5%)
FPG mmol/L (mg/dL)	5.10 ± 0.61 (91.8 ± 10.9)	4.88 ± 0.50 (87.9 ± 8.99)	5.36 ± 0.72 (96.5 ± 12.9)
1-h PG mmol/L (mg/dL)	8.89 ± 2.31 (160 ± 41.7)	6.39 ± 1.88 (115 ± 33.8)	10.26 ± 2.05 (185 ± 37)
2-h PG mmol/L (mg/dL)	7.11 ± 2.44 (128 ± 44)	5.77 ± 1.67 (104 ± 30)	11.65 ± 2.49 (210 ± 44.8)
HbA1c (%)	5.24 ± 0.368%	5.17 ± 0.355%	5.40 ± 0.637%

1-h PG, 1 h plasma glucose; NGT, normal glucose tolarence; IGT, impaired glucose tolarence; FPG, fasting plasma glucose; 2-h PG, 2 h plasma glucose; HbA1c, glycated hemoglobin

^*a*^Monophasic curve shape is defined as a continuous rise in plasma glucose concentration reaching a single peak, followed by a subsequent decrease of more than 0.25 mmol/L (4.5 mg/dL)

^*b*^Biphasic shape was characterized by an initial rise and fall in glucose concentration concentrations, followed by a secondary increase of greater than 0.25 mmol/L (4.5 mg/dL) after the initial decline

^c^A continuous or rising pattern was defined as a curve without identifiable peaks, exhibiting a persistent upward trajectory in glucose levels throughout the test duration

**Fig. 3:**
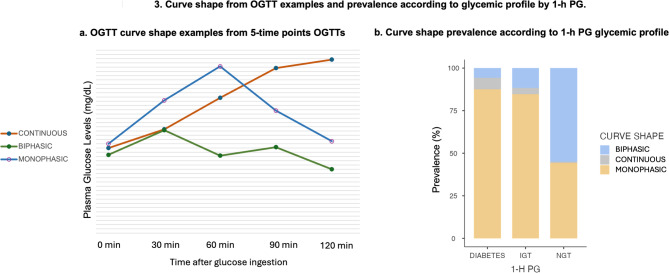
Curve shape from OGTT examples and prevalence according to glycemic profile by 1-h PG. **A**. OGTT curves showing monophasic, biphasic, and continuous rise patterns from 5-point OGTTs. **B**. Distribution of curve morphologies according to 1-h PG glycemic profile, showing significantly higher monophasic prevalence in T2DM and IH, while biphasic curves predominate in NGT (χ^2^ = 278; P < 0.001). 1-h PG, 1-h plasma glucose; *NGT* normal glucose tolerance; *IH* intermediate hyperglycemia

A significant association was observed between curve morphology and glycemic status determined by 1-h PG (χ^2^ = 278; df = 4; p < 0.001) (Fig. 3). The monophasic pattern was predominant among individuals with T2DM (87%) and IH (85%), whereas the biphasic pattern was more frequently observed in normoglycemic individuals.

When stratified by sex, the association between glycemic status and curve shape was significant in both sexes (Women: χ^2^ = 184.2, df = 4, p < 0.001; Men: χ^2^ = 96.2, df = 4, p < 0.001). For the monophasic curve, women showed a higher proportion of normal glucose tolerance (49.6%) compared to men (40.4%), while men had more abnormal outcomes (59.6% vs. 50.4%). In the biphasic curve, the majority were classified as normal, slightly higher in women (89.9%) than in men (88.7%). Although less common, the continuous curve was strongly associated with dysglycemia in both sexes, affecting 73.7% of women and 90.0% of men.

## Discussion

This study evaluates the 1-h PG diagnostic performance, compared with other diagnostic methods, in a not well previously explored population: Brazilian non-pregnant individuals. Our findings demonstrate that the 1-h PG threshold of ≥ 155 mg/dL can detect a significantly greater number of individuals with IH than other diagnostic methods in this population cohort. Among the 844 individuals who underwent both 1-h PG during OGTT and HbA1c testing, 22.3% were diagnosed with IH by 1-h PG but not by HbA1c, while HbA1c alone detected only 4.7% with IH who were not identified by 1-h PG. This indicates that approximately 1 in 5 individuals with pre-diabetes were classified as normoglycemic by HbA1c. Furthermore, 1-h PG demonstrated greater efficacy than HbA1c combined with FPG in detecting individuals at high glycemic risk (p < 0.001). Similarly, Abdul-Ghani et al. described 1-h PG as a stronger predictor of future T2DM than HbA1c, and Jagannathan et al. showed a stronger correlation between 1-h PG ≥ 155 mg/dL with β-cell dysfunction and insulin resistance compared to HbA1c ≥ 5.7% (p < 0.05). [9, 12] Additionally, Peddinti et al. showed that HbA1c alone or in combination with clinical risk factors has lower diagnostic acurance in detecting hyperglycemia than 1-h PG. [21].

Tábak et al. reported that alterations in glycemic concentrations may precede the diagnosis of type 2 diabetes (T2DM) by up to six years during the OGTT, thus increasing the potential for the early detection of dysglycemia in high-risk individuals. This fact allows earlier interventions to prevent the progression of glycemic dysfunction and its subsequent complications [22].

When comparing 1-h PG with 2-h PG, moderate agreement was observed between the tests (κ = 0.479; p < 0.001), with 1-h PG being more sensitive in detecting individuals with IH and T2DM, identifying 302 (16.8%) more individuals with dysglycemia than 2-h PG. In contrast, 2-h PG identified only 3.2% of individuals with IH who were not diagnosed by 1-h PG, demonstrating that relying solely on the conventional 2-h PG parameter results in greater diagnostic loss compared to 1-h PG alone. Guerreiro et al. also found moderate agreement between the two tests (κ = 0.405; p < 0.001) in diagnosing obese individuals with IH. In the same study, 1-h PG detected 24.8% more individuals with IH than 2-h PG. [23].

In a longitudinal study following Native Americans for 6–10 years, Ha et al. showed that 1-h PG indeed detects IH on average 1.6 years earlier and T2DM year earlier, findings that were reinforced by Bergman et al., who reaffirmed the higher sensitivity and predictive power of 1-h PG compared to 2-h PG and FPG for the development of T2DM. [10, 13] In another prospective study, Pareek et al. found that elevated 1-h PG values were associated with the incidence of diabetes (HR 3.40; p < 0.001) as well as a higher association with cardiovascular complications and mortality, consistent with other studies [24–27]. Monea et al. reported that when compared to NGT individuals, those with 1-h PG ≥ 155 mg/dL and NGT were more likely associated with cardiac autonomic dysfunction, Rong et al. showed its superiority over 2-h PG in predicting cardiovascular events and mortality, and Fiorentino et al. demonstrated its capacity to detect individuals at increased risk of chronic kidney disease beyond independently of other cardiometabolic risk factors. [28–30] Additionally, compared to FPG, 1-h PG showed higher sensitivity for diagnosis in this study, consistent with findings from the Botnia Study. [31].

It was observed that 15.7% of Brazilian individuals with NGT at the 2-hour time point presented 1-h PG ≥ 8.6 mmol/L (155 mg/dL), while this proportion ranges from 10.8% to 42.6% in other population-based studies. [10] This metabolic profile has been characterized as being associated with greater β-cell dysfunction. [14, 32] Additionally, data from a cross-sectional and longitudinal cohort study of American Indians demonstrated that 1-h PG has a predictive capacity for diabetic retinopathy risk similar to 2-h PG. [17] Beyond that, cost-effectiveness analyses have showed that screening prediabetes with the 1-h plasma glucose test is not only clinically effective and economically advantageous—reducing long-term diabetes-related complications and overall healthcare costs despite higher initial expenditures—but also more practical and feasible in routine settings compared with the 2-h PG. [10, 33].

In the studied population, the monophasic glucose curve was more commonly observed among female participants and was associated with higher mean age, FPG, 1-h PG, 2-h PG, and HbA1c levels compared to individuals with a biphasic curve. A statistically significant association was demonstrated between the 1-h PG glycemic profile and the curve pattern. Individuals with IH and type 2 diabetes (T2DM) were more frequently associated with the monophasic curve, while those with NGT exhibited a stronger correlation with the biphasic curve. Worse glycemic profiles were correlated with the continuous curve pattern.

Consistent with these findings, de Andrade Mesquita et al. found that Brazilian individuals with a monophasic curve presented with higher fasting glucose, 1-h PG (p < 0.001), and 2-h PG (p = 0.028) levels. Furthermore, monophasic pattern in this population was more strongly associated with prediabetes and metabolic syndrome. Similar trend have been confirmed in other cohorts; for example, Cheng et al. demonstrated that, in Chinese individuals, the monophasic curve was linked to older age and worse glycemic profiles. [18, 34] Furthermore, Fischer et al. found that monophasic curves was more associated with higher concentrations of fasting GLP-1. In parallel, Kim et al. observed that obese youths with monophasic curves have higher concentrations of C-peptide, free fatty acid and insulin. [35, 36] Additionally, Abdul-Ghani et al. suggested that the glucose curve pattern derived from the OGTT can be used to assess the risk of developing T2DM. [37] Finally, it was well described by Sabolic et al., Tura et al. and Cheng et al. that the monophasic curve shape morphology from OGTT is the pattern most associated with impaired beta cell β-cell function. [18, 38, 39].

Our limitations are primarily related to its cross-sectional design. We did not evaluate longitudinally whether Brazilian individuals diagnosed with IH by 1-h PG were indeed more likely to develop type 2 diabetes (T2DM) and comorbidities associated with hyperglycemia. Additionally, we did not assess the time interval required for individuals classified as IH solely based on 1-h PG to be subsequently diagnosed by other methods. Similarly, we did not investigate whether individuals with NGT but exhibiting a monophasic curve pattern were more likely to develop dysglycemia compared to those with a biphasic curve.

In summary, this study demonstrated that, for the Brazilian population, the thresholds of 1-h PG ≥ 8.6 mmol/L (155 mg/dL) for IH and 1-h PG ≥ 11.6 mmol/L (209 mg/dL) for type 2 diabetes (T2DM) identify more individuals than the parameters used by traditional diagnostic methods. Thus, 1-h PG serves as an important tool to detect a greater number of individuals with dysglycemia, allowing for earlier interventions to prevent disease progression. Additionally, it was observed that individuals with a monophasic curve pattern are more frequently associated with altered glycemic profiles (IH or diabetes) and elevated glucose levels. However, further longitudinal studies are necessary to evaluate the metabolic and clinical outcomes in this population.

## Data Availability

The data that support the findings of this study are available from Emilio Ribas Medicina Diagnostica Ltda but restrictions apply to the availability of these data, which were used under license for the current study, and so are not publicly available. Data are however available from the authors upon reasonable request and with permission of Emilio Ribas Medicina Diagnostica Ltda.

## Abbreviations

1-h PG: 1-Hour plasma glucose
OGTT: Oral glucose tolerance test
FPG: Fasting plasma glucose
2-h PG: 2-Hour plasma glucose
Hb1Ac: Glycated hemoglobin
IH: Intermediate hyperglycemia
T2DM: Type 2 diabetes mellitus
DM: Diabetes mellitus
ADA: American diabetes association
SBD: Brazilian diabetes society
IFG: Impaired fasting glucose
IGT: Impaired glucose tolerance
WHO: World health organization
IDF: International diabetes federation
